# Phenotypic Trajectories From Acute to Stable Phase in Heart Failure With Preserved Ejection Fraction: Insights From the PURSUIT‐HFpEF Study

**DOI:** 10.1161/JAHA.124.037567

**Published:** 2025-02-03

**Authors:** Yuki Matsuoka, Yohei Sotomi, Daisaku Nakatani, Katsuki Okada, Akihiro Sunaga, Hirota Kida, Taiki Sato, Daisuke Sakamoto, Tetsuhisa Kitamura, Sho Komukai, Masahiro Seo, Masamichi Yano, Takaharu Hayashi, Akito Nakagawa, Yusuke Nakagawa, Shunsuke Tamaki, Yoshio Yasumura, Takahisa Yamada, Shungo Hikoso, Yasushi Sakata, the OCVC‐Heart Failure Investigators

**Affiliations:** ^1^ Department of Cardiovascular Medicine Osaka University Graduate School of Medicine Osaka Japan; ^2^ Department of Medical Informatics Osaka University Graduate School of Medicine Osaka Japan; ^3^ Department of Social and Environmental Medicine Osaka University Graduate School of Medicine Osaka Japan; ^4^ Division of Biomedical Statistics, Department of Integrated Medicine, Graduate School of Medicine Osaka University Osaka Japan; ^5^ Division of Cardiology Osaka General Medical Center Osaka Japan; ^6^ Division of Cardiology Osaka Rosai Hospital Osaka Japan; ^7^ Cardiovascular Division Osaka Police Hospital Osaka Japan; ^8^ Division of Cardiology Amagasaki Chuo Hospital Amagasaki Hyogo Japan; ^9^ Shoushoukai Healthcare Corporation, Nakagawa Clinic Osaka Japan; ^10^ Division of Cardiology Kawanishi City Medical Center Amagasaki Hyogo Japan; ^11^ Department of Cardiology Rinku General Medical Center Osaka Japan; ^12^ Department of Cardiology, Pulmonology, Hypertension and Nephrology Ehime University Graduate School of Medicine Ehime Japan; ^13^ Department of Cardiovascular Medicine Nara Medical University Kashihara Japan

**Keywords:** HFpEF, latent class analysis, machine learning, phenotyping, Heart Failure

## Abstract

**Background:**

Using machine learning for the phenotyping of patients with heart failure with preserved ejection fraction (HFpEF) has emerged as a novel approach to understanding the pathophysiology and stratifying the patients. Our objective is to perform phenotyping of patients with HFpEF in stable phase and to investigate the phenotypic trajectory from acute worsening phase to stable phase.

**Methods:**

The present study is a post hoc analysis of the PURSUIT‐HFpEF (Prospective Multicenter Observational Study of Patients with Heart Failure with Preserved Ejection Fraction) study. We applied the latent class analysis to the discharge data of patients hospitalized for acute decompensated heart failure.

**Results:**

We finally included patient data of 1100 cases and 63 features in the latent class analysis. All patients were subclassified into 5 phenogroups as follows: Phenotype 1, characterized by better renal function and lower NT‐proBNP (N‐terminal pro‐B‐type natriuretic peptide) level [N=325 (29.5%)]; Phenotype 2, higher blood pressure, sinus rhythm, and poor renal function. [N=242 (22.0%)]; Phenotype 3, higher prevalence of atrial fibrillation, higher tricuspid pressure gradient, and lower tricuspid annular plane systolic excursion [N=214 (19.5%)]; Phenotype 4, higher C‐reactive protein level and higher tricuspid pressure gradient [N=245 (22.3%)]; and Phenotype 5, poor nutritional status, poor renal function, and higher NT‐proBNP level [N=74 (6.7%)]. A particular phenotype observed at the time of discharge was correlated with a distinct phenotype of acute worsening.

**Conclusions:**

We identified 5 distinct stable phase phenotypes of the patients with HFpEF from the data at discharge. A specific phenotype at discharge was associated with a particular phenotype of acute worsening. This grouping can be a basis for future precision medicine of patients with HFpEF.

**Registration:**

URL: https://www.umin.ac.jp/ctr/; Unique identifier: UMIN000021831.

**Nonstandard Abbreviation and Acronym**
HFpEF: heart failure with preserved ejection fraction

**Clinical Perspective**

**What Is New?**

- We applied the latent class analysis to the data of patients with heart failure with preserved ejection fraction at discharge and successfully identified 5 distinct stable‐phase phenotypes in a real‐world East Asian cohort of heart failure with preserved ejection fraction.
- A particular phenotype observed at the time of discharge was correlated with a distinct phenotype of acute phase.

**What Are the Clinical Implications?**

- The specific pattern in the phenotypic trajectory has provided important insights for identifying therapeutic intervention targets in patients with heart failure with preserved ejection fraction.

The incidence of heart failure with preserved ejection fraction (HFpEF) is escalating and is projected to exceed that of heart failure with reduced ejection fraction in the near future.^1^ Presently, the pharmacological interventions for HFpEF are predominantly limited to diuretics and sodium‐glucose cotransporter‐2 inhibitors, unlike the more comprehensive and established therapeutic options available for heart failure with reduced ejection fraction.^2^, ^3^, ^4^ This discrepancy in treatment effectiveness is attributed not only to the lack of universally accepted diagnostic criteria as well as variation in inclusion criteria in clinical trials but also to the inherent diversity in the pathophysiological mechanisms of HFpEF. Given this diversity, a personalized approach to identify treatment targets tailored to each pathophysiology is essential for establishing effective therapies for HFpEF.

Using machine learning for the phenotyping of patients with HFpEF has emerged as a novel approach to understanding the pathophysiology and stratifying this patient cohort.^5^, ^6^ In our previous research, we applied latent class analysis to phenotype patients experiencing acute HFpEF, effectively classifying them into 4 unique phenotypes based on data from hospital admissions: rhythm trouble, ventricular‐arterial uncoupling, low output and systemic congestion, and systemic failure.^7^ This investigation diverged from earlier studies in Western populations, which predominantly concentrated on data from stable phases.^5^, ^6^ Moreover, the characteristics of patients with HFpEF exhibit variations between Japan and Western nations.^8^, ^9^ In this study, our objective is to perform phenotyping analysis of patients with HFpEF in Japan using discharge data, aiming to phenotype patients during the stable phase. Additionally, this study endeavors to elucidate the pathogenesis of HFpEF and identify potential therapeutic interventions by assessing phenotypic trajectories from admission to discharge.

## METHODS

Our study data will not be made available to other researchers for purposes of reproducing the results because of institutional review board restrictions.

### Study Design and Settings

The present study is a post‐hoc analysis of the database of the Prospective mUlticenteR obServational stUdy of patIenTs with Heart Failure with preserved Ejection Fraction (PURSUIT‐HFpEF) study. The PURSUIT‐HFpEF study is a prospective, multi‐referral center, observational study in which collaborating hospitals in Osaka record clinical data of patients with acute decompensated HFpEF [UMIN‐CTR ID: UMIN000021831].^10^ Consecutive patients who were hospitalized for acute decompensated heart failure and preserved ejection fraction (≥50%) were prospectively registered and agreed to be followed up for collection of outcome data. Acute decompensated heart failure was diagnosed on the basis of the following criteria: (1) clinical symptoms and signs according to the Framingham Heart Study criteria^11^; and (2) serum NT‐proBNP (N‐terminal pro‐B‐type natriuretic peptide) level of ≥400 pg/mL or brain natriuretic peptide (BNP) level of ≥100 pg/mL. All patients provided written informed consent for participation in this study. The study protocol was approved by the ethics committee of each participating hospital. This study conformed to the ethical guidelines outlined in the Declaration of Helsinki. Details of the data collection have been described elsewhere.^12^, ^13^ In brief, basic patient characteristics, echocardiography, laboratory tests, and lists of medications were obtained on admission, at discharge, and at each annual follow‐up time point.

### Clinical Outcomes

In the PURSUIT‐HFpEF study, all patients were followed up in each hospital after discharge. Clinical follow‐up data were obtained by dedicated coordinators and investigators by direct contact with patients and their physicians at the hospital or in an outpatient setting or by a telephone interview with their families or by mail. The primary end point was a composite of all‐cause death and heart failure hospitalization. The secondary end points were individual components of the primary end point.

### Statistical Analysis

The machine‐learning‐based unsupervised cluster analysis and other statistical analyses were all performed using R software (version 4.3.1; R Foundation for Statistical Computing, Vienna, Austria). A *P* value <0.05 was considered statistically significant.

We applied the machine‐learning‐based unsupervised cluster analysis, latent class analysis, to the PURSUIT‐HFpEF data set.^6^, ^7^, ^14^ A total of 149 variables at hospital discharge were considered as primary candidates for the latent class analysis. Covariates included a wide range of domains including patient demographics, clinical variables, laboratory data, and echocardiographic parameters. A total of 112 variables were finally included in the present analysis after exclusion of factors with ≥20% missing data. Missing data were imputed by random forest imputation using “missForest” package prior to variable selection and clustering due to the benefits of random forest imputation over multivariate imputation by chained equations (“MICE”) and mixture models.^15^ Variables with a correlation coefficient >0.6 were filtered keeping the variable that was most clinically relevant and informative. As a result, 63 continuous and categorical variables were used in the final phenotyping analysis (Table 1). We used the “VarSelLCM” package in R 4.3.1 to try between 2 to 10 clusters to identify the optimal number of clusters and most relevant discriminatory variables. In general, the optimal number of phenogroups within the cohort using the model‐based clustering can be determined with optimization of the Bayesian Information Criterion. The Bayesian Information Criterion introduces a penalty term for the number or parameters in the model to avoid the overfitting of the model created. The optimal number of the clusters is the model with the lowest Bayesian Information Criterion value. We computed the discriminative power of each variable, which was defined as the logarithm of the ratio between the probability that the variable is relevant for clustering and the variable is irrelevant for clustering.

**Table 1:** Patients' Background in 5 Phenogroups

	Phenotype 1 “Low comorbidity”	Phenotype 2 “Hypertension & CKD”	Phenotype 3 “AF & concomitant RHF”	Phenotype 4 “Systemic inflammation & concomitant RHF”	Phenotype 5 “Malnutrition & CKD”	*P* value	Missing, %
n	325	242	214	245	74		
Age, y	83.00 [77.00–87.00]	82.00 [76.25–86.00]	82.00 [77.00–86.00]	84.00 [78.00–88.00]	78.00 [71.25–83.00]	<0.001	0
Male	112 (34.5)	94 (38.8)	126 (58.9)	118 (48.2)	46 (62.2)	<0.001	0
Body mass index, kg/m^2^	21.08 [18.43–24.07]	22.47 [19.64–25.38]	21.10 [18.75–23.40]	22.06 [19.50–24.84]	21.26 [18.56–23.89]	<0.001	0.9
Prior heart failure hospitalization	70 (22.2)	48 (20.0)	74 (35.2)	56 (23.3)	19 (26.0)	0.002	2
Hypertension	251 (77.2)	235 (97.5)	172 (80.4)	212 (87.2)	60 (81.1)	<0.001	0.3
Diabetes	29 (9.0)	145 (60.2)	72 (33.8)	76 (31.3)	40 (54.1)	<0.001	0.7
Coronary artery disease	43 (13.4)	59 (24.9)	34 (16.0)	37 (15.2)	17 (23.6)	0.003	1.2
Chronic kidney disease	67 (20.8)	134 (55.8)	102 (47.7)	96 (39.7)	43 (58.1)	<0.001	0.7
Pacemaker implantation	25 (7.7)	22 (9.1)	31 (14.5)	16 (6.5)	2 (2.7)	0.007	0.1
Systolic blood pressure, mm Hg	115.00 [105.00–126.00]	128.00 [112.25–141.00]	116.00 [104.00–126.00]	118.00 [105.00–132.00]	120.00 [109.25–131.50]	<0.001	0
Diastolic blood pressure, mm Hg	115.00 [105.00–126.00]	128.00 [112.25–141.00]	116.00 [104.00–126.00]	118.00 [105.00–132.00]	120.00 [109.25–131.50]	<0.001	0
Heart rate, bpm	70.00 [62.00–77.00]	69.00 [60.25–78.00]	68.00 [60.00–78.00]	71.00 [63.00–80.00]	74.00 [63.25–82.00]	0.07	0
Atrial fibrillation at discharge	118 (36.4)	54 (22.3)	120 (56.1)	102 (41.6)	18 (24.3)	<0.001	0.1
6‐minute walk distance, m	255.00 [184.00–347.50]	250.00 [175.00–330.00]	271.50 [160.00–360.00]	218.00 [122.00–306.00]	285.00 [200.00–354.00]	0.008	47.6
EQ‐5D‐5L score	0.78 [0.60–0.90]	0.78 [0.61–0.89]	0.82 [0.71–1.00]	0.71 [0.49–0.88]	0.78 [0.49–0.89]	<0.001	15
Cardiothoracic ratio, %	55.00 [51.00–59.92]	54.30 [50.40–58.30]	58.00 [53.00–63.90]	56.20 [52.00–62.00]	54.70 [50.00–60.00]	<0.001	1.2
CHADS_2_ score						<0.001	1.6
1	19 (6.0)	1 (0.4)	2 (0.9)	6 (2.5)	4 (5.4)		
2	74 (23.3)	17 (7.2)	42 (19.8)	26 (10.8)	16 (21.6)		
3	181 (56.9)	85 (35.9)	101 (47.6)	121 (50.2)	25 (33.8)		
4	19 (6.0)	93 (39.2)	44 (20.8)	51 (21.2)	20 (27.0)		
5	22 (6.9)	21 (8.9)	10 (4.7)	27 (11.2)	3 (4.1)		
6	3 (0.9)	20 (8.4)	13 (6.1)	10 (4.1)	6 (8.1)		
New York Heart Association functional class						0.011	1.4
I	134 (42.0)	97 (40.4)	77 (36.2)	65 (27.1)	26 (35.6)		
II	166 (52.0)	127 (52.9)	126 (59.2)	151 (62.9)	37 (50.7)		
III	19 (6.0)	15 (6.2)	10 (4.7)	22 (9.2)	9 (12.3)		
IV	0 (0.0)	1 (0.4)	0 (0.0)	2 (0.8)	1 (1.4)		
Clinical frailty scale						0.011	0.3
1	17 (5.3)	6 (2.5)	4 (1.9)	5 (2.0)	7 (9.5)		
2	75 (23.2)	58 (24.1)	51 (23.8)	36 (14.7)	12 (16.2)		
3	84 (26.0)	64 (26.6)	57 (26.6)	69 (28.2)	19 (25.7)		
4	54 (16.7)	38 (15.8)	52 (24.3)	47 (19.2)	14 (18.9)		
5	22 (6.8)	29 (12.0)	18 (8.4)	22 (9.0)	2 (2.7)		
6	42 (13.0)	23 (9.5)	18 (8.4)	34 (13.9)	11 (14.9)		
7	25 (7.7)	21 (8.7)	14 (6.5)	28 (11.4)	8 (10.8)		
8	4 (1.2)	2 (0.8)	0 (0.0)	4 (1.6)	1 (1.4)		
Trigger of hospitalization for acute decompensated heart failure							
Infection	45 (13.8)	46 (19.0)	21 (9.8)	56 (22.9)	19 (25.7)	<0.001	0
Arrythmia	120 (36.9)	44 (18.2)	53 (24.8)	73 (29.8)	17 (23.0)	<0.001	0
Uncontrollable hypertension	43 (13.2)	46 (19.0)	20 (9.3)	39 (15.9)	18 (24.3)	0.006	0

Data are expressed as median [interquartile range] or number (percentage).

After we identified the optimal clusters, we assessed the differences in patient demographics, clinical variables, laboratory data, echocardiographic parameters, and medications at discharge. Data are presented with listwise deletion. In the radar charts, continuous variables were displayed as means of Z‐standardized values and categorical variables were displayed as proportion. We referred to data from our previous reports for comparison with the clustering at admission.^7^, ^16^, ^17^ Categorical variables are expressed as counts (percentages) and compared with the chi‐squared test. Continuous variables are expressed as median [interquartile range] and compared using a 1‐way ANOVA (or Kruskal–Wallis test, when appropriate).^14^ Risk of the end points across the phenogroups was assessed in a time‐to‐first‐event fashion with the Kaplan–Meier method and compared with the log‐rank test. We illustrated radar charts in which continuous variables were displayed as means of Z‐standardized values and categorical variables were displayed as proportion. The phenotypic trajectory from the acute worsening phase to the stable phase was investigated with reference to our previous studies on acute‐phase phenotyping.^7^, ^16^, ^17^

### Systematic Review

Machine learning‐based approach has been used in previous studies. We conducted a systematic review about the machine learning‐based clustering for patients with HFpEF (PROSPERO: CRD42024542803). We searched Pubmed and Web of Science for all article published up to March 2024. Studies were included if they defined new subgroups in patients with HFpEF using clustering analysis methods by unsupervised algorithms.

## RESULTS

**Figure 1:**
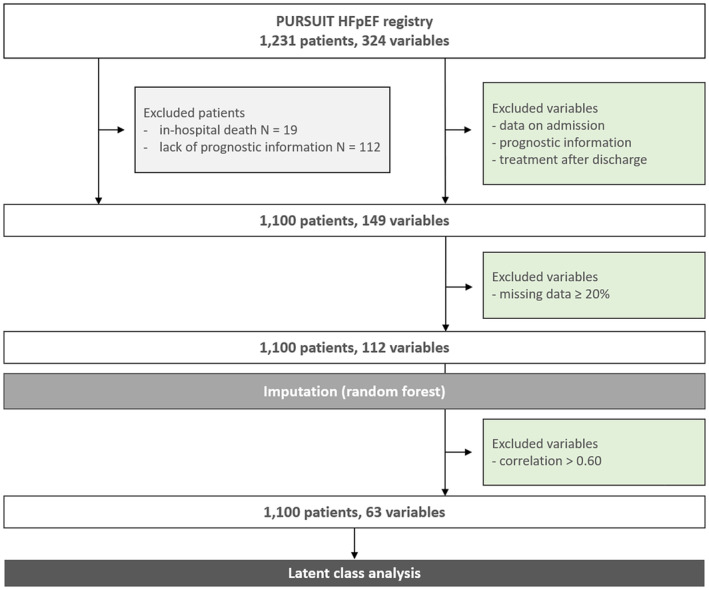
Study flowchart. A total of 1231 patients were enrolled in the PURSUIT HFpEF registry. Nineteen patients died during hospitalization and there were 112 patients who did not have the prognostic information. We had 324 variables in this registry and excluded 175 variables such as data on admission, prognostic information, and information of treatments after discharge. In addition, we excluded variables with significant missingness (≥20%) and high degree of correlation (correlation coefficient >0.60). A total of 1100 patients and 63 variables were finally included in the machine learning‐based unsupervised cluster analysis (latent class analysis).

**Figure 2:**
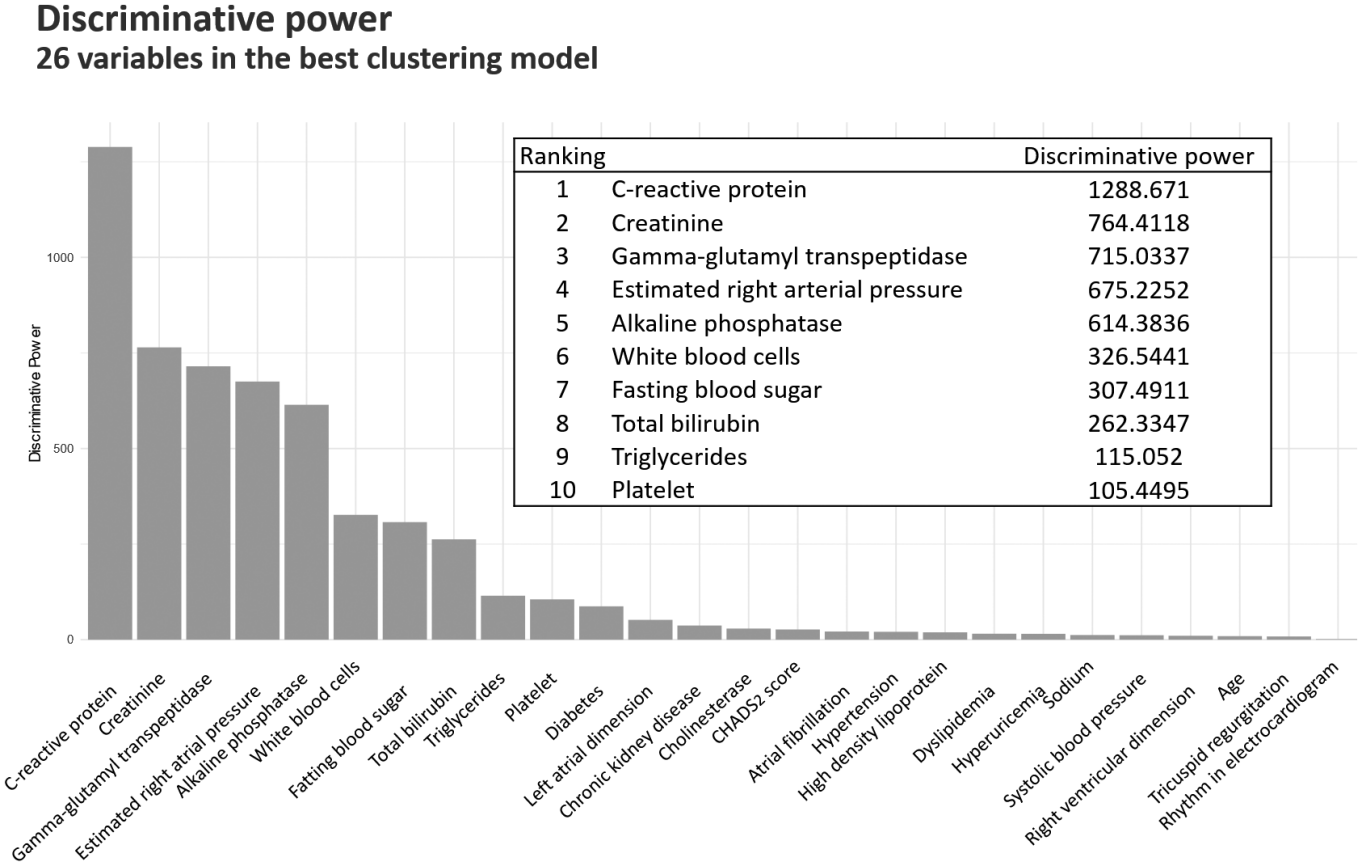
Discriminative power ranking. Discriminative power of the 26 variables selected by latent class analysis is shown as a bar graph. We computed the discriminative power of each variable which was defined as the logarithm of the ratio between the probability that the variable is relevant for clustering and the variable is irrelevant for clustering.

We used patient data of 1231 cases enrolled in the PURSUIT‐HFpEF registry between 2016 and 2022. Patients with in‐hospital death (N=19) and who lacked the prognostic information (N=112) were excluded. A total of 1100 patients were finally analyzed in this study (Figure 1). Baseline characteristics, laboratory tests, and echocardiographic data at hospital discharge (149 features) were the primary candidates for the clustering analysis. After excluding features with significant missingness (≥20%) and high degree of correlation (r>0.6), a total of 63 features (Table S1) were finally included in the machine learning‐based unsupervised cluster analysis (latent class analysis). In this analysis, we observed a trend where the Bayesian Information Criterion decreased with an increasing number of clusters. However, with more groups, the clustering becomes less practical for everyday clinical use and interpreting the characteristics of each group becomes challenging. Using the elbow method, we determined that clustering into 5 groups strikes a balance, offering an optimal number of clusters for both practical application and interpretability (Figure S1). The latent class analysis selected 26 variables for the optimal model. These variables were summarized in Figure 2 with their discriminative power of top 10. The most discriminatory variable was C‐reactive protein, followed by creatinine, gamma‐glutamyl transpeptidase, estimated right atrial pressure, alkaline phosphatase, and white blood cells. The probability of misclassification is illustrated in Figure S2, showing that the misclassification rate seems negligible.

### Phenogroups

**Figure 3:**
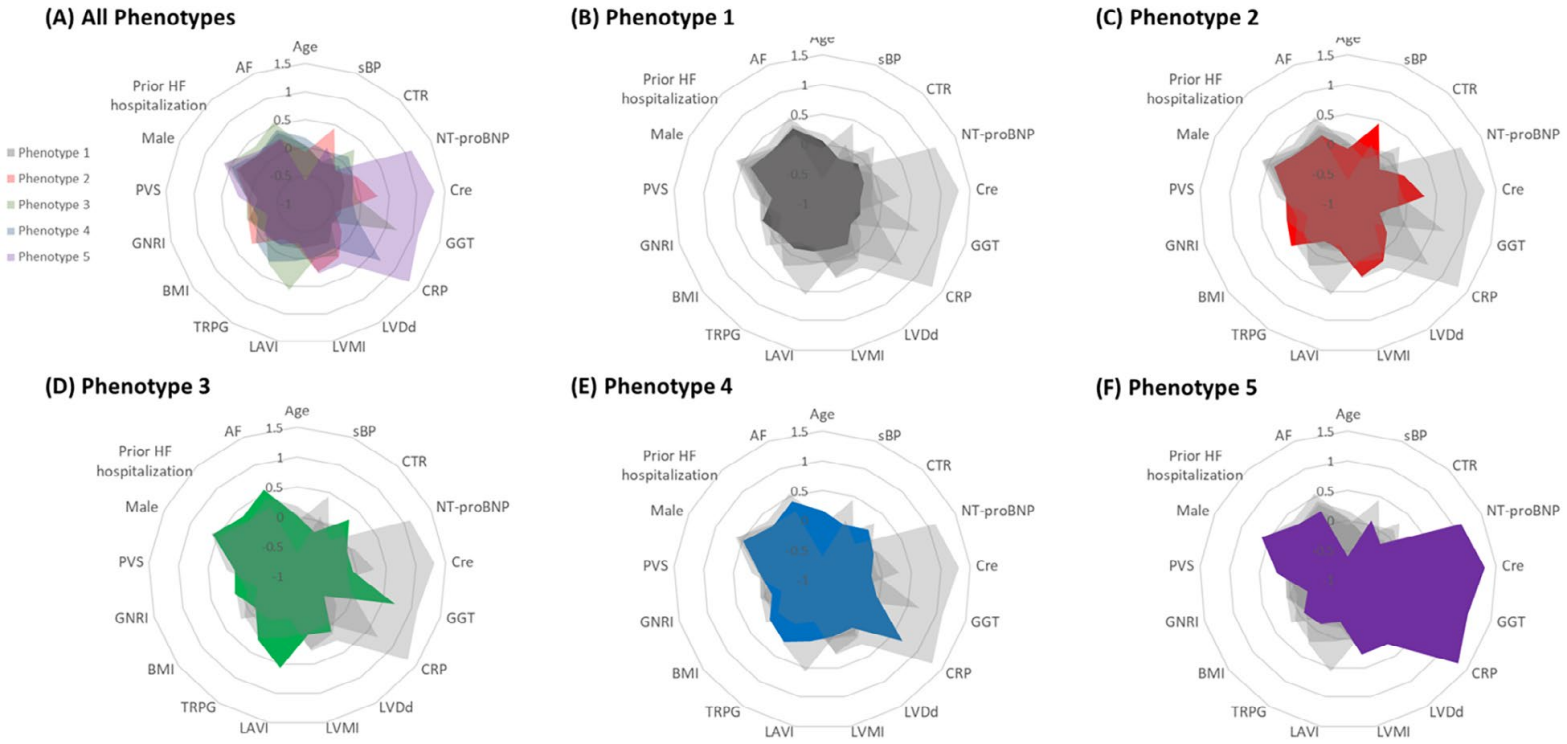
Clinical features in 5 phenogroups. Relative differences in the clinical features among the 5 phenogroups are illustrated in the radar chart. **A**, Overlay of all 5 phenotypes (1–5) for overall comparison. **B** through **F**, Emphasized profiles of Phenotypes 1 through 5, respectively, highlighting their distinct characteristics. Continuous variables are displayed as means of Z‐standardized values and categorical variables are displayed as proportion. AF indicates atrial fibrillation; BMI, body mass index; GGT, gamma‐glutamyl transpeptidase; CRP, C‐reactive protein; CTR, cardio thoracic ratio; GNRI, geriatric nutritional risk index; HF, heart failure; LAVI, left atrial volume index; LVDd, left ventricular diastolic dimension; LVMI, left ventricular mass index; PVS, plasma volume status; sBP, systolic blood pressure; and TRPG, transtricuspid pressure gradient.

All patients subclassified into 5 phenogroups as follows: Phenotype 1, N=325 (29.5%); Phenotype 2, N=242 (22.0%); Phenotype 3, N=214 (19.5%); Phenotype 4, N=245 (22.3%); and Phenotype 5, N=74 (6.7%). Patients' characteristics across the 5 groups are summarized in Table 1. Laboratory and echocardiographic data are shown in Table 2.^18^ Relative differences in the clinical features among the 5 phenogroups are illustrated in Figure 3.

**Table 2:** Laboratory and Echocardiographic Data in 5 Phenogroups

	Phenotype 1 “Low comorbidity”	Phenotype 2 “Hypertension & CKD”	Phenotype 3 “AF & concomitant RHF”	Phenotype 4 “Systemic inflammation & concomitant RHF”	Phenotype 5 “Malnutrition & CKD”	*P* value	Missing
n	325	242	214	245	74		
NT‐proBNP, pg/mL	731.00 [365.80, 1490.00]	1210.00 [469.00, 2790.00]	1145.00 [566.02, 2460.00]	1305.00 [574.50, 2664.00]	2540.00 [1013.50, 6781.00]	<0.001	15.3
White blood cells, *10^3^/μL	5.20 [4.35, 6.30]	5.80 [4.80, 7.15]	4.60 [3.92, 5.20]	6.30 [5.00, 7.60]	6.60 [5.43, 9.18]	<0.001	0.2
Hemoglobin, g/dL	11.80 [10.65, 13.30]	10.75 [9.62, 12.07]	11.50 [10.30, 13.10]	11.00 [10.00, 12.30]	11.40 [9.57, 12.50]	<0.001	0.2
Platelet, *10^4^/μL	21.10 [17.40, 25.95]	22.55 [18.02, 28.80]	17.40 [14.62, 20.45]	24.00 [18.40, 30.80]	20.30 [15.93, 28.77]	<0.001	0.2
Creatinine, mg/dL	1.00 [0.80, 1.10]	1.40 [1.00, 2.20]	1.20 [0.90, 1.60]	1.10 [0.80, 1.50]	1.45 [1.00, 3.68]	<0.001	0.3
Estimated glomerular filtration rate, mL/min per 1.73 m^2^	47.70 [37.50, 58.25]	33.20 [20.00, 49.00]	41.10 [29.80, 53.80]	43.85 [29.85, 57.70]	33.20 [12.80, 54.40]	<0.001	2
Uric acid, mg/dL	6.40 [5.40, 7.70]	6.70 [5.50, 8.00]	6.60 [5.50, 7.80]	7.00 [5.50, 8.50]	6.80 [5.25, 8.05]	0.222	4.1
Total protein, g/dL	6.60 [6.20, 7.10]	6.60 [6.15, 7.10]	6.70 [6.20, 7.20]	6.70 [6.30, 7.18]	6.60 [6.10, 7.20]	0.693	4.1
Albumin, g/dL	3.50 [3.30, 3.70]	3.30 [3.10, 3.70]	3.60 [3.30, 3.80]	3.20 [2.90, 3.60]	3.20 [2.80, 3.50]	<0.001	2.8
Sodium, mEq/L	140.00 [138.00, 142.00]	140.00 [137.00, 141.00]	139.00 [137.00, 141.00]	139.00 [137.00, 141.00]	137.00 [134.00, 139.75]	<0.001	0.3
Potassium, mEq/L	4.30 [3.95, 4.60]	4.30 [3.90, 4.60]	4.30 [4.00, 4.60]	4.30 [3.90, 4.60]	4.10 [3.80, 4.57]	0.211	0.2
Alkaline phosphatase, IU/L	221.00 [186.00, 263.00]	228.00 [189.00, 277.25]	293.50 [217.00, 380.00]	256.00 [210.00, 316.00]	298.50 [203.00, 554.00]	<0.001	4.4
γ‐glutamyl transferase, IU/L	26.50 [18.00, 37.75]	21.00 [15.00, 31.00]	77.50 [38.75, 119.50]	36.00 [23.75, 62.25]	52.00 [23.00, 148.00]	<0.001	8.4
Cholinesterase, IU/L	224.00 [193.00, 272.00]	219.00 [179.00, 281.25]	187.00 [158.00, 217.25]	195.50 [151.75, 235.75]	172.00 [149.00, 232.00]	<0.001	19.3
Total bilirubin, mg/dL	0.60 [0.50, 0.80]	0.50 [0.40, 0.60]	0.70 [0.50, 1.00]	0.60 [0.40, 0.70]	0.60 [0.40, 1.28]	<0.001	0.9
C‐reactive protein, mg/dL	0.17 [0.08, 0.31]	0.40 [0.15, 0.85]	0.14 [0.06, 0.23]	1.50 [0.60, 2.70]	1.31 [0.42, 3.89]	<0.001	1.3
Total cholesterol, mg/dL	165.00 [146.00, 189.00]	156.00 [135.25, 182.75]	154.50 [132.75, 173.25]	152.00 [131.00, 173.00]	151.00 [127.75, 184.25]	<0.001	13.9
Triglyceride, mg/dL	93.00 [70.00, 116.00]	117.00 [91.25, 153.00]	81.00 [63.00, 103.00]	99.50 [71.00, 131.00]	105.00 [79.00, 182.00]	<0.001	12
Low density lipoprotein cholesterol, mg/dL	100.00 [83.00, 118.50]	86.50 [71.00, 109.00]	85.50 [68.25, 103.50]	89.00 [72.00, 109.00]	82.00 [66.00, 112.00]	<0.001	13.8
High density lipoprotein cholesterol, mg/dL	47.50 [40.00, 56.00]	39.00 [34.00, 47.50]	47.00 [39.00, 58.00]	41.00 [34.00, 48.00]	39.00 [30.00, 47.00]	<0.001	13.9
Fasting blood sugar, mg/dL	47.50 [40.00, 56.00]	39.00 [34.00, 47.50]	47.00 [39.00, 58.00]	41.00 [34.00, 48.00]	39.00 [30.00, 47.00]	<0.001	13.9
Geriatric Nutritional Risk index	92.62 [85.40, 99.47]	92.05 [84.67, 101.32]	92.16 [87.02, 99.08]	90.83 [82.87, 97.67]	91.51 [79.64, 96.02]	0.041	3.6
Controlling nutritional status score	2.00 [1.00, 4.00]	3.00 [2.00, 5.00]	3.00 [2.00, 5.00]	4.00 [3.00, 6.00]	5.00 [3.00, 6.00]	<0.001	15.9
Plasma volume status,^18^ %	8.93 [−1.21, 16.48]	11.68 [3.00, 20.23]	11.47 [3.08, 20.00]	12.45 [2.19, 19.18]	12.62 [1.66, 22.07]	0.023	0.5
Left ventricular ejection fraction (Teichholz), %	64.10 [59.10, 69.15]	64.65 [59.45, 70.00]	62.35 [56.80, 68.30]	66.20 [60.50, 72.10]	62.10 [55.08, 66.97]	<0.001	6.5
Left ventricular end‐diastolic dimension, mm	44.00 [40.00, 48.73]	46.00 [42.00, 50.00]	47.00 [42.00, 51.00]	45.20 [41.00, 49.50]	47.85 [43.10, 52.88]	<0.001	6.5
Left atrial dimension, mm	42.00 [37.00, 47.00]	44.00 [40.00, 47.00]	48.00 [41.00, 53.00]	45.00 [40.00, 50.00]	42.50 [38.00, 47.00]	<0.001	7.1
E/e′ (septal)	14.10 [10.50, 19.22]	16.80 [12.80, 21.10]	14.40 [11.35, 18.65]	14.60 [11.95, 19.95]	15.15 [11.70, 20.95]	0.002	9.9
Right ventricular dimension, mm	31.50 [27.00, 35.00]	32.00 [28.00, 35.00]	34.95 [30.55, 39.00]	32.55 [27.58, 37.00]	32.00 [27.50, 37.00]	<0.001	19.3
Tricuspid annular plane systolic excursion, cm	17.30 [15.00, 20.00]	18.20 [16.00, 21.00]	16.70 [13.00, 19.90]	17.25 [14.50, 20.00]	17.90 [14.90, 20.60]	0.009	15.3
Trans tricuspid pressure gradient, mm Hg	26.00 [21.25, 31.00]	24.40 [21.00, 29.30]	29.00 [23.00, 36.75]	29.00 [23.90, 34.40]	24.50 [19.12, 31.00]	<0.001	13.8
Left ventricular mass index, g/m^2^	95.42 [78.09, 113.30]	107.57 [91.48, 133.86]	102.22 [85.02, 124.92]	101.17 [82.63, 123.21]	114.14 [95.43, 127.83]	<0.001	7.2
Estimated right atrial pressure, mm Hg	3.00 [3.00, 3.00]	3.00 [3.00, 3.00]	8.00 [3.00, 8.00]	8.00 [3.00, 8.00]	3.00 [3.00, 8.00]	<0.001	12.3
Aortic regurgitation						0.104	3.8
None	117 (36.9)	106 (47.1)	85 (40.1)	84 (36.1)	32 (45.1)		
Trace	77 (24.3)	62 (27.6)	48 (22.6)	55 (23.6)	14 (19.7)		
Mild	96 (30.3)	40 (17.8)	68 (32.1)	76 (32.6)	21 (29.6)		
Moderate	25 (7.9)	16 (7.1)	11 (5.2)	18 (7.7)	4 (5.6)		
Severe	2 (0.6)	1 (0.4)	0 (0.0)	0 (0.0)	0 (0.0)		
Mitral regurgitation						0.027	3.8
None	33 (10.4)	27 (12.0)	18 (8.5)	20 (8.6)	9 (12.7)		
Trace	102 (32.2)	84 (37.3)	57 (26.9)	70 (30.0)	29 (40.8)		
Mild	138 (43.5)	82 (36.4)	89 (42.0)	104 (44.6)	25 (35.2)		
Moderate	43 (13.6)	32 (14.2)	44 (20.8)	38 (16.3)	6 (8.5)		
Severe	1 (0.3)	0 (0.0)	4 (1.9)	1 (0.4)	2 (2.8)		
Tricuspid regurgitation						<0.001	3.8
None	21 (6.6)	21 (9.3)	12 (5.7)	20 (8.6)	7 (9.9)		
Trace	102 (32.2)	105 (46.7)	50 (23.6)	60 (25.8)	32 (45.1)		
Mild	133 (42.0)	82 (36.4)	83 (39.2)	84 (36.1)	22 (31.0)		
Moderate	54 (17.0)	15 (6.7)	56 (26.4)	56 (24.0)	9 (12.7)		
Severe	7 (2.2)	2 (0.9)	11 (5.2)	13 (5.6)	1 (1.4)		

Data are expressed as median [interquartile range] or number (percentage). NT‐proBNP indicates N‐terminal pro‐B‐type natriuretic peptide.

We named these phenotypes “Low comorbidity,” “Hypertension & chronic kidney disease (CKD),” “Atrial fibrillation (AF) & concomitant right heart failure (RHF),” “Systemic inflammation & concomitant RHF,” and “Malnutrition & CKD,” respectively. Patients in Phenotype 1, “Low comorbidity,” had lower plasma volume status, better renal function, lower NT‐proBNP level, and better nutritional status than the other phenotypes. Phenotype 2, “Hypertension & CKD,” was characterized by higher systolic blood pressure, sinus rhythm at discharge, poorer renal function, and more comorbidities such as diabetes. Patients in Phenotype 3, “AF & concomitant RHF,” showed the higher prevalence of AF, largest cardiothoracic ratio on chest x‐ray, higher tricuspid pressure gradient, and lower tricuspid annular plane systolic excursion on echocardiography at discharge. The proportion of patients who experienced heart failure hospitalization before was higher than those in other phenotypes. Phenotype 4, “Systemic inflammation & concomitant RHF” was characterized by higher C‐reactive protein level, and elevated tricuspid pressure gradient. This phenotype showed the worst 6‐minute walk distance. Phenotype 5, “Malnutrition & CKD,” included patients who had poorer nutritional status and poor renal function. Patients in this phenotype had higher NT‐proBNP level.

Oral medications at hospital discharge are tabulated in Table S2. Antiarrhythmic drugs were more used in Phenotype 1. Antiplatelet drugs and calcium channel blockers were frequently used in Phenotype 2 and 5. Diuretics use was more common in the Phenotype 3 and 4.

### Association of Phenotypes With Clinical Outcomes

**Figure 4:**
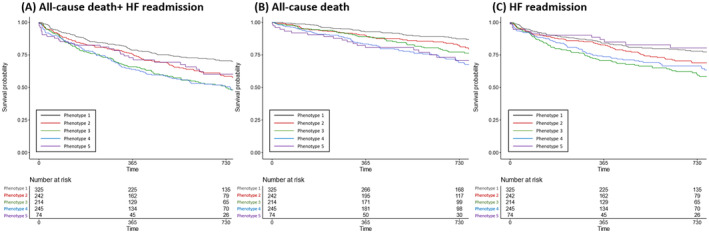
Clinical outcomes. Survival analysis was conducted with Kaplan–Meier methods for a composite of all‐cause death and heart failure readmission (**A**), all‐cause death (**B**), and heart failure readmission (**C**). HF indicates heart failure.

Incidences of clinical outcomes are summarized in Table S3. Median follow‐up duration was 733.5 [interquartile range 397.8, 1108.0] days. Kaplan–Meier analysis showed a significant difference in the primary end point between the phenogroups (Figure 4). Patients in Phenotypes 3 and 4 had the worse prognosis, while Phenotype 1 showed the best prognosis.

### Phenotypic Trajectory of Patients With HFpEF

**Figure 5:**
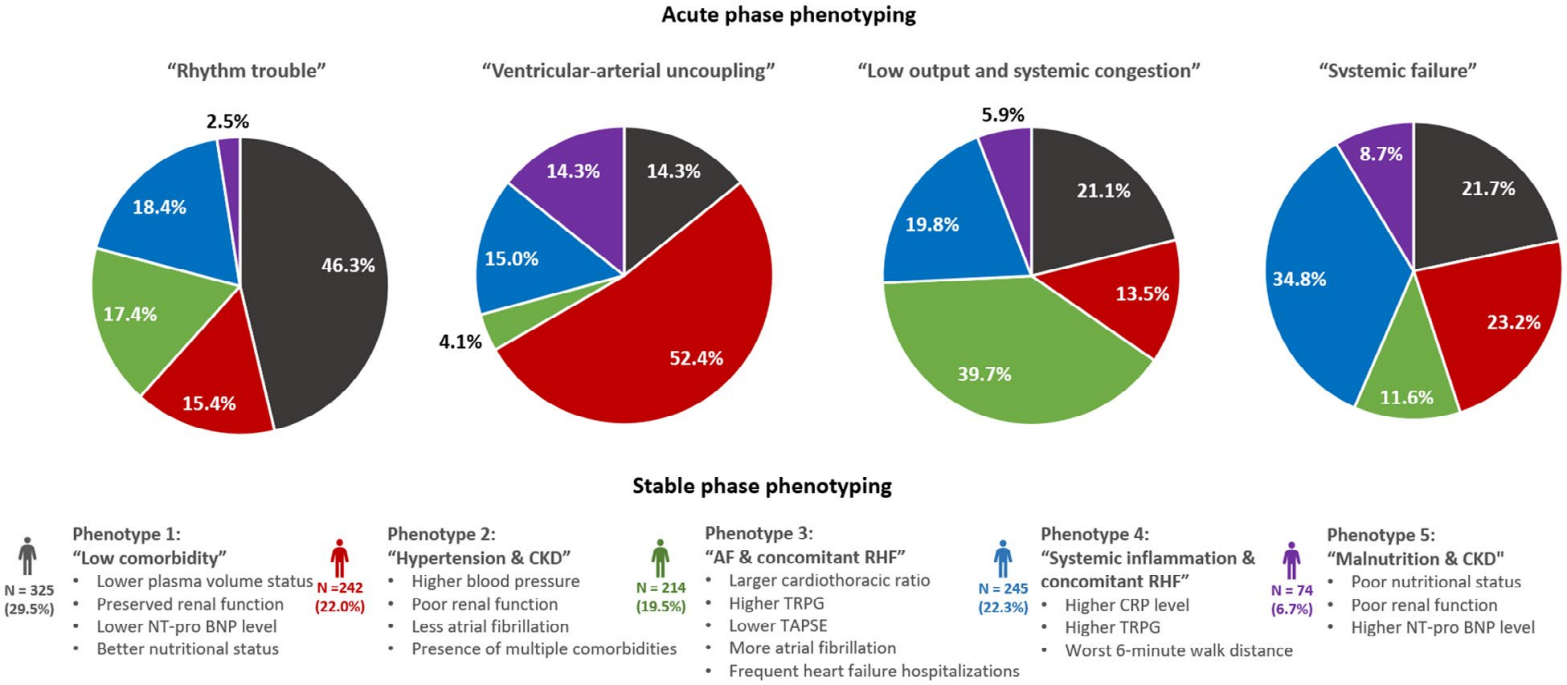
Phenotypic trajectory from hospital admission (acute phase) to discharge (stable phase). This figure comprises 4 pie charts, each representing the percentage distribution of stable‐phase phenotypes (at hospital discharge) across acute‐phase phenotypes (at hospital admission). BNP indicates brain natriuretic peptide; TRPG, transtricuspid pressure gradient; TAPSE, tricuspid annular plane systolic excursion; and CRP, C‐reactive protein.

In our previous research, we applied latent class analysis to the acute‐phase data and classified them into 4 unique acute‐phase phenotypes: rhythm trouble, ventricular‐arterial uncoupling, low output and systemic congestion, and systemic failure.^7^ Our current study explores the evolution of these phenotypes from the time of hospital admission to discharge (Figure 5). The phenotype characterized by rhythm trouble predominantly transitioned to Phenotype 1, “Low comorbidity.” Those identified with ventricular‐arterial uncoupling primarily evolved into Phenotype 2, “Hypertension & CKD.” The group with low output and systemic congestion mainly diverged into Phenotype 3, “AF & concomitant RHF,” and Phenotype 4, “Systemic inflammation & concomitant RHF.” Lastly, the systemic failure group primarily transitioned into Phenotype 4, “Systemic inflammation & concomitant RHF.” We assessed long‐term outcomes stratified by the trajectories from acute‐phase phenotype to stable‐phase phenotype (Figure S3). While prognosis varied by stable‐phase phenotype, it also differed within each stable‐phase phenotype depending on the acute‐phase phenotype.

### Systematic Review

Twenty‐eight studies were finally eligible in our review. We summarized the brief methods and the results of the review in Figure S4 and Table S4. The most frequently used method was hierarchical clustering. In most studies, patients were classified into 3 to 4 clusters. Variables included in the analyses mainly consisted of patient background and laboratory data and, in some studies, unique variables such as biomarkers.

## DISCUSSION

The main findings of the present study were summarized as follows: (1) We demonstrated that the machine‐learning‐based unsupervised clustering approach by using the data at discharge successfully identified stable‐phase subphenotypes in a real‐world East Asian cohort of HFpEF. This is the first report which focused on phenotyping in both acute‐worsening and stable phase of the same cohort of HFpEF; (2) The 5 phenogroups identified in the current study had distinct baseline characteristics and clinical outcomes; (3) A particular phenotype observed at the time of discharge was correlated with a distinct phenotype of acute phase.

Machine‐learning‐based approach has been used in previous studies. We conducted the systematic review about the machine learning‐based clustering for patients with HFpEF (Table S4). We used latent class analysis in this study although the most common approach was hierarchical clustering. Latent class analysis offers significant advantages over hierarchical clustering, primarily through its model‐based approach that provides a probabilistic framework for data analysis. This method enables probabilistic class assignments, effectively capturing uncertainties about data belonging to multiple classes. Additionally, latent class analysis is well‐suited for handling heterogeneous data types and offers clear interpretation of classes through parameter estimation.^19^ In most studies, patients were classified into 3 to 4 clusters. As indicated in Table S4, our sample size and candidate variables included in the clustering model was relatively large among the studies, which might have enabled more detailed analysis and resulted in the larger number of phenogroups as compared with the previous studies.

Our group previously conducted the machine‐learning‐based clustering by using the data on hospital admission of the same registry and successfully identified the phenotypes of acute worsening phase.^7^ Additionally, the analysis based on the phenotyping of acute worsening phase suggested that the effects of medication on prognosis seemed to be different among these phenotypes.^17^, ^20^ As such, the machine‐learning‐based approach for HFpEF seems to be potentially useful in improving the prognosis of HFpEF. On the other hand, this previous analysis only included patients who needed hospitalization for acute decompensated heart failure. The other past studies which used the machine learning‐based clustering of HFpEF applied the approach for the data of stable phase or outpatients (Table S4). We have therefore established a phenotyping method that can be used for stable patients by using a similar approach for data at the time of discharge, which would allow us to potentially extend the findings to outpatients and compare the current findings to the previous Western data.

Comparing the phenotypes in the present and previous studies, relatively young and low BNP phenotypes, like Phenotype 1 in the present study, were commonly found.^21^ As shown in another Japanese study, it was notable that obesity was not disproportionately prevalent, and a higher incidence of elderly patients was observed in our East Asian cohort in comparison with Western cohorts.^22^, ^23^ The most discriminative feature was C‐reactive protein, followed by creatinine, gamma‐glutamyl transpeptidase, estimated right atrial pressure, and alkaline phosphatase in this study. This finding was dissimilar to the findings from the major studies.^5^, ^6^ In the analysis from TOPCAT, the most discriminative feature was glucose level, followed by total bilirubin, diabetes, statin use, and body mass index.^6^ In the I‐PRESERVE study, 11 clinical features, age, sex, body mass index, AF, coronary artery disease, diabetes, dyslipidemia, valvular disease, alcohol use, estimated glomerular filtration rate, and hematocrit were used.^5^ This result might be attributed to not only the different variables included in each study but also the difference of patient characteristics of HFpEF between East Asia and the Western countries.^8^

### Features of Phenotypes and Phenotypic Transition From Acute to Convalescent

We named Phenotype 1 “Low comorbidity” because patients who belonged to this phenotype had fewer comorbidities and better prognosis. Phenotype 2 was named “Hypertension & CKD.” Patients included in this phenotype had higher systolic blood pressure, less AF, and higher prevalence of multiple comorbidities. Phenotype 3 was named “AF & concomitant RHF.” In this phenotype, the pressure of the right heart system was significantly higher and the right ventricular function was lower than the other phenotypes. Echocardiographic findings suggestive of fluid retention and a history of hospitalization for heart failure were common. Phenotype 4 was named “Systemic inflammation & concomitant RHF”. Patients in this phenotype had higher C‐reactive protein level. There were findings of congestion seconded by Phenotype 3. Phenotype 5 was named “Malnutrition & CKD”. Patients in this phenotype had poor nutritional status, poor renal function, and higher NT‐proBNP level. Patients in Phenotype 5 had a lower frequency of heart failure readmissions but a higher rate of all‐cause death compared with the other phenotypes (Table S3). It is speculated that extracardiac factors may have a significant prognostic impact on Phenotype 5.

In this study, we investigated the phenotypic trajectory from acute phase to stable phase. The half of patients with worsening due to rhythm trouble transitioned into Phenotype 1. Worsening triggered by arrhythmia was more common in patients with HFpEF with relatively better background and less comorbidities. The half of patients who experienced “ventricular‐arterial uncoupling” as the acute‐phase phenotype primarily evolved into Phenotype 2. About 40% of patients whose phenotype of acute phase was “Low output and systemic congestion” mainly diverged into Phenotype 3. About 35% of patients with “systemic failure” in acute phase transitioned into Phenotype 4 at discharge. For any acute‐phase phenotypes, a small number of patients transitioned into Phenotype 5 at the stable phase. These results may indicate probability distribution of worsening pattern of HFpEF. Diverse mechanisms of acute decompensation can exist depending on the patient's condition. One specific phenotype of acute phase does not always singularly translate into a specific stable‐phase phenotype. Nonetheless, the predominance of a specific mechanism represented by a relatively frequent trajectory in each phenotype suggests the potential for identifying therapeutic intervention targets. Prospective studies predicated on this hypothesis are anticipated to pave the way for the development of precision medicine tailored to patients with HFpEF.

### Study Limitations

Several limitations should be acknowledged. First, generalizability of the current findings to the other regions, races, and ethnicities is limited because of the differing health care systems and economic status in Japan compared with other countries. Second, the significant missingness in several features [37/149 features (24.8%)] did not allow us to include the features in the latent class analysis, resulting in the selection bias. Third, this registry included HFpEF mimics such as hypertrophic cardiomyopathy and cardiac amyloidosis, as excluding only confirmed mimics could have introduced bias due to variations in their evaluation across facilities and the likelihood that some cases were undiagnosed. Fourth, this study included only hospitalized patients, limiting the generalizability of our phenotyping to outpatients although this study focused on the stable phase. Further investigation is needed to determine whether this phenotyping applies to outpatients as well. Lastly, statistical validation analysis of this type of clustering analysis is genuinely impossible because the latent class analysis is categorized as unsupervised machine learning. The validity of the present clustering model should be tested with clinical validity and relevance with targeted treatments in future prospective studies.

## CONCLUSIONS

By applying latent class analysis to the stable‐phase data of patients with HFpEF, we identified 5 distinct phenotypes characterized by unique patient features. We found that a specific phenotype at discharge was associated with a particular phenotype of acute phase. Establishment of phenotype‐specific treatment with this machine‐learning model could be a basis of future precision medicine of patients with HFpEF.

## Sources of Funding

This work was funded by Roche Diagnostics K.K. and Fuji Film Toyama Chemical Co. Ltd.

## Disclosures

Dr Sotomi has received grants from Roche Diagnostics, FUJIFILM Toyama Chemical, TOA EIYO, Bristol‐Myers Squibb, Biosense Webster, Abbott Medical Japan, and NIPRO, and personal fees from Abiomed, AstraZeneca, Amgen Astellas BioPharma, Biosensors, Boehringer Ingelheim, Bristol‐Myers Squibb, Abbott Medical Japan, Boston Scientific Japan, Bayer, Daiichi Sankyo, Novartis, TERUMO, Medtronic, and Pfizer Pharmaceuticals. Dr Hikoso has received personal fees from Daiichi Sankyo Company, Bayer, Astellas Pharma, Pfizer Pharmaceuticals, Novartis Pharmaceuticals, Kowa Company, Otsuka Pharmaceutical, AstraZeneca, Eli Lilly Japan, Ono Pharmaceutical, TOA EIYO, Kyowa Kirin, and Boehringer Ingelheim Japan, including speaking and lecture fees. Dr Hikoso has received grants from Roche Diagnostics, FUJIFILM Toyama Chemical, TOA EIYO, and Bristol Myers Squibb. Dr Nakatani has received personal fees from Roche Diagnostics. Y. Sakata has received personal fees from Otsuka Pharmaceutical, Ono Pharmaceutical, Daiichi Sankyo, Mitsubishi Tanabe Pharma Corporation, AstraZeneca K.K. and Actelion Pharmaceuticals, and grants from Roche Diagnostic, FUJIFILM Toyama Chemical, Bristol‐Myers Squibb, Co, Biosense Webster, Inc., Abbott Medical Japan, Otsuka Pharmaceutical, Daiichi Sankyo Company, Mitsubishi Tanabe Pharma Corporation, Astellas Pharma, Kowa Company, Boehringer Ingelheim Japan, and Biotronik. The remaining authors have no disclosures to report.

## Supporting information

Data S1
Tables S1–S4
Figures S1–S4
