# Supplementary material for: Phenotypic Trajectories From Acute to Stable Phase in Heart Failure With Preserved Ejection Fraction: Insights From the PURSUIT‐HFpEF Study
Source: J Am Heart Assoc. 2025 Feb 3;14(3):e037567. doi: 10.1161/JAHA.124.037567 (PMC12074776; doi:10.1161/JAHA.124.037567)

Supplemental Appendix

The OCVC-Heart Failure Investigators

Chair: Yasushi Sakata, Department of Cardiovascular Medicine, Osaka University Graduate School of Medicine, 2–2 Yamada-oka, Suita 565–0871, Japan.

Study manager: Yohei Sotomi (Chief), Daisaku Nakatani, Katsuki Okada, Tomoharu Dohi, Akihiro Sunaga, Hirota Kida, Taiki Sato, Yuki Matsuoka, and Daisuke Sakamoto; Department of Cardiovascular Medicine, Osaka University Graduate School of Medicine, Suita, Japan.

Investigators: Masahiro Seo, Tetsuya Watanabe, and Takahisa Yamada, Osaka General Medical Center, Osaka, Japan; Takaharu Hayashi and Yoshiharu Higuchi, Osaka Police Hospital, Osaka, Japan; Masaharu Masuda, Mitsutoshi Asai, and Toshiaki Mano, Kansai Rosai Hospital, Amagasaki, Japan; Hisakazu Fuji, Kobe Ekisaikai Hospital, Kobe, Japan; Shunsuke Tamaki, Daisaku Masuda, Ryu Shutta, and Shizuya Yamashita, Rinku General Medical Center, Izumisano, Japan; Masami Sairyo and Yusuke Nakagawa, Kawanichi City Medical Center, Kawanishi, Japan; Haruhiko Abe, Yasunori Ueda, and Yasushi Matsumura, National Hospital Organization Osaka National Hospital, Osaka, Japan; Kunihiko Nagai, Ikeda Municipal Hospital, Ikeda, Japan; Masamichi Yano, Masami Nishino, and Jun Tanouchi, Osaka Rosai Hospital, Sakai, Japan; Yoh Arita and, Nobuyuki Ogasawara, Japan Community Health Care Organization Osaka Hospital, Osaka, Japan; Takamaru Ishizu, Minoru Ichikawa and Yuzuru Takano, Higashiosaka City Medical Center, Higashiosaka, Japan; Eisai Rin, Kawachi General Hospital, Higashiosaka, Japan; Yukinori Shinoda, Koichi Tachibana and Shiro Hoshida, Yao Municipal Hospital, Yao, Japan; Masahiro Izumi, Kinki Central Hospital, Itami, Japan; Hiroyoshi Yamamoto and Hiroyasu Kato, Japan Community Health Care Organization, Osaka Minato Central Hospital, Osaka, Japan; Kazuhiro Nakatani and Yuji Yasuga, Sumitomo Hospital, Osaka, Japan; Mayu Nishio and Keiji Hirooka, Saiseikai Senri Hospital, Suita, Japan; Takahiro Yoshimura, Kazunori Kashiwase and Shinji Hasegawa, National Hospital Organization Osaka Minami Medical Center, Kawachinagano, Japan; Akihiro Tani, Kano General Hospital, Osaka, Japan; Yasushi Okumoto, Kinan Hospital, Tanabe, Japan; Yasunaka Makino, Hyogo Prefectural Nishinomiya Hospital, Nishinomiya, Japan; Toshinari Onishi and Katsuomi Iwakura, Sakurabashi Watanabe Hospital, Osaka, Japan; Yoshiyuki Kijima, Japan Community Health Care Organization, Hoshigaoka Medical Center, Hirakata, Japan; Takashi Kitao, Minoh City Hospital, Minoh, Japan; Masashi Fujita, Osaka International Cancer Institute, Osaka, Japan; Koichiro Harada, Suita Municipal Hospital, Suita, Japan; Masahiro Kumada and Osamu Nakagawa, Toyonaka Municipal Hospital, Toyonaka, Japan; Ryo Araki and Takayuki Yamada, Otemae Hospital, Osaka, Japan;

Akito Nakagawa and Yoshio Yasumura, Amagasaki Chuo Hospital, Amagasaki, Japan; and Daisuke Sakamoto, Yuki Matsuoka, Taiki Sato, Akihiro Sunaga, Hirota Kida, Yohei Sotomi, Tomoharu Dohi, Yasuhiro Akazawa, Kei Nakamoto, Katsuki Okada, Fusako Sera, Hidetaka Kioka, Tomohito Ohtani, Toshihiro Takeda, Daisaku Nakatani, Shungo Hikoso, and Yasushi Sakata, Osaka University Graduate School of Medicine, Suita, Japan.

Table S1. Variables included latent class analysis

Number			
1	Age	33	Dyslipidemia
2	Systolic blood pressure	34	Smoking
3	Diastolic blood pressure	35	Drinking history
4	Heart rate	36	History of hyperuricemia
5	Score of quality of life	37	Hypertrophic cardiomyopathy
6	Cardiothoracic ratio	38	Secondary cardiomyopathy
7	White blood cells	39	Coronary artery disease
8	Platelet	40	Coronary artery bypass grafting
9	Creatinine	41	Heart surgery
10	Uric acid	42	Sick sinus syndrome
11	Total protein	43	Atrioventricular block
12	Sodium	44	Pericardial disease
13	Potassium	45	Pacemaker implantation
14	Alkaline phosphatase	46	Sleep apnea syndrome
15	Gamma-glutamyl transpeptidase	47	Aortic aneurysm
16	Cholinesterase	48	Chronic kidney disease
17	Total bilirubin	49	Liver dysfunction
18	C-reactive protein	50	Malignant tumor
19	Triglycerides	51	Family history of heart failure
20	High-density lipoprotein	52	Bleeding history
21	Low-density lipoprotein	53	Home oxygen therapy
22	Fasting blood sugar	54	New York Heart Association functional class
23	Left atrial dimension	55	Rhythm on electrocardiogram
24	Right ventricular dimension	56	Atrial fibrillation
25	Tricuspid annular plane systolic excursion	57	Critical arrythmia
26	CHADS2 score	58	Aortic regurgitation
27	Body mass index	59	Mitral regurgitation
28	Estimated right atrial pressure	60	Tricuspid regurgitation
29	Left ventricular mass index	61	Aortic stenosis
30	Prior heart failure hospitalization	62	Mitral stenosis
31	Hypertension	63	Pericardiac effusion
32	Diabetes		

Table S2. Oral medication at discharge in 5 phenogroups

	Phenotype 1 "Low comorbidity"	Phenotype 2 "Hypertension & CKD"	Phenotype 3 "AF & concomitant RHF"	Phenotype 4 "Systemic inflammation & concomitant RHF"	Phenotype 5 "Malnutrition & CKD"	P value	Missing
n	325	242	214	245	74		
Antiplatelet therapy	62 (19.1)	102 (42.1)	44 (20.6)	77 (31.6)	29 (39.2)	<0.001	0.1
Angiotensin converting enzyme inhibitor or angiotensin receptor blocker	181 (55.7)	134 (55.4)	112 (52.3)	130 (53.1)	36 (48.6)	0.785	0
Calcium channel blocker	121 (37.2)	165 (68.2)	94 (43.9)	115 (47.1)	39 (52.7)	<0.001	0.1
Beta blocker	182 (56.0)	135 (55.8)	107 (50.0)	137 (56.1)	48 (64.9)	0.255	0.1
Diuretics	245 (75.4)	197 (81.4)	188 (87.9)	213 (86.9)	55 (74.3)	<0.001	0
Mineral corticoid receptor antagonist	123 (37.8)	81 (33.5)	107 (50.0)	95 (38.8)	27 (36.5)	0.006	0
Statin	93 (28.6)	112 (46.3)	66 (30.8)	78 (32.0)	22 (29.7)	<0.001	0.1

Sodium-glucose cotransporter-2 inhibitor	4 (1.2)	22 (9.1)	11 (5.1)	11 (4.5)	11 (14.9)	<0.001	0.2
Glucagon-like peptide-1 receptor antagonist	0 (0.0)	4 (1.7)	2 (0.9)	3 (1.2)	1 (1.4)	0.297	0.1
Anticoagulants	201 (61.8)	99 (40.9)	166 (77.6)	144 (58.8)	39 (52.7)	<0.001	0
Antiarrhythmic drug	43 (13.2)	11 (4.5)	18 (8.4)	19 (7.8)	4 (5.4)	0.005	0.1

Data are expressed as number (percentage).

Table S3. Clinical outcomes

	Phenotype 1 "Low comorbidity"	Phenotype 2 "Hypertension & CKD"	Phenotype 3 "AF & concomitant RHF"	Phenotype 4 "Systemic inflammation & concomitant RHF"	Phenotype 5 “Malnutrition & CKD"	P value (log-rank)
n	325	242	214	245	74	
All-cause death or HF readmission	117/325, 18.6/100 person-year	118/242, 28.0/100 person-year	123/214, 35.7/100 person-year	137/245, 36.9/100 person-year	33/74, 25.5/100 person-year	<0.001
All-cause death	68/325, 9.1/100 person-year	68/242, 12.3/100 person-year	67/214, 14.3/100 person-year	91/245, 18.8/100 person-year	21/74, 13.9/100 person-year	<0.001
HF readmission	77/325, 12.2/100 person-year	78/242, 18.5/100 person-year	89/214, 25.8/100 person-year	85/245, 22.9/100 person-year	17/74, 13.1/100 person-year	<0.001

Abbreviation: HF, heart failure.

Table S4. Systematic review of previous machine-learning cluster analyses of HFpEF patients

Cohort name	Method	Data	Variable number	Setting	Patient number	Number of clusters	Country	Year	Reference
The EHOP CDW of RUHC	Spectral clustering	Patient background, Laboratory data, Echocardiography, Electrocardiogram	26	Stable phase	2500	4	France	2024	Pierre-Jean M, et al. Eur Heart J Open 2024;4(1):oead133.
DIAMOND-HFpEF	K-means	Patient background, Echocardiography, Biomarker	25	Stable phase	136	3	UK	2024	Dattani A, et al. BMC Cardiovasc Disord 2024;24(1):94.
ASIAN-HF	K-means	Echocardiography	14	Stable phase	888	5	Multinational (Asia)	2023	Teramoto K, et al. JACC Asia 3 (5):739-751.
WET- HF2	Latent class analysis	Patient background, Laboratory data	17	Stable phase	826	3	Japan	2023	Nakamaru R, et al. J Am Heart Assoc 12 (3):e027689.
2019 National Readmission Database	Mixture model based	Patient background	20	Stable phase	1066	4	US	2023	Mohebi R, et al. Int J Cardiol 378:71-76.
NARA-HF	Mixture model based	Patient background, Laboratory data, Echocardiography	24	Stable phase	365	3	Japan	2023	Kyodo A, et al. ESC Heart Fail 10 (3):2019-2030.
PURSUIT-HFpEF	Latent class analysis	Patient background, Laboratory data, Echocardiography	32	Acute phase	1095	4	Japan	2022	Sotomi Y, et al. Heart 108 (19):1553-1561.
ASCEND-HF	Hierarchical clustering	Patient background, Laboratory data	39	Acute phase	812	4	Multinational	2022	Murray E, et al. Am Heart J 254:112-121.
Hôpital Européen Georges-Pompidou	Hierarchical clustering	Patient background, Laboratory data, Echocardiography	14	Stable phase	928	3	France	2022	Fayol A, et al. ESC Heart Fail 9 (1):519-530.
TOPCAT	Latent class analysis	Patient background, Laboratory data	10	Stable phase	623	2	US	2022	Dong B, et al. BMC Med 20 (1):423.
Framingham Heart Study	Spectral clustering	mRNA, miRNA, DNA	45174	Stable phase	125	3	US	2021	Wu Y, et al. Comput Struct Biotechnol J 19:1567-1578.
BIOSTAT-CHF	Hierarchical clustering	Biomarker	363	Stable phase	429	4	UK	2021	Woolley R, et al. Eur J Heart Fail 23 (6):983-991.
SwedeHF	Latent class analysis	Patient background, Laboratory data	10	Stable phase	6909	5	Sweden	2021	Uijl A, et al. Eur J Heart Fail 23 (6):973-982.
University of British Columbia	Partitioning Around Medoids	Patient background, Laboratory data, Echocardiography	47	Stable phase	196	6	Canada	2021	Nouraei H, et al. Int J Cardiol 331:138-143.
Shanghai Ninth People's Hospital	Hierarchical clustering	Patient background, Laboratory data, Echocardiography	11	Stable phase	970	3	China	2021	Gu J, et al. Int J Cardiol 323:148-154.
OptimEx-Clin	Hierarchical clustering	Patient background, Laboratory data, Echocardiography	33	Stable phase	176	3	Europe	2021	Gevaert A, et al. Front Physiol 12:757268.
Medicare Advantage Prescription Drug or commercial healthcare plan	Hierarchical clustering	Patient background	20	Stable phase	1515	3	US	2021	Casebeer A, et al. J Cardiovasc Med (Hagerstown) 22 (1):45-52.

Cohort name	Method	Data	Variable number	Setting	Patient number	Number of clusters	Country	Year	Reference
MEDIA-DHF	K-means	Biomarker	349	Stable phase	392	2	Europe	2020	Stienen S, et al. Biomarkers 25 (2):201-211.
TOPCAT	Mixture model based	Patient background, Laboratory data, Echocardiography	20	Stable phase	1767	3	US	2020	Segar M, et al. Eur J Heart Fail 22 (1):148-158.
KaRen	Hierarchical clustering	Patient background, Laboratory data, Echocardiography	55	Stable phase	356	3	Europe	2020	Schrub F, et al. Arch Cardiovasc Dis 113 (6-7):381-390.
KaRen	Mixture model based	Patient background, Laboratory data, Echocardiography	43	Stable phase	320	6	Europe	2020	Hedman Å K, et al. Heart 106 (5):342-349.
TOPCAT	Latent class analysis	Patient background, Laboratory data	8	Stable phase	3342	3	US	2020	Cohen J B, et al. JACC Heart Fail 8 (3):172-184.
DICUMAP	Partitioning Around Medoids	Patient background, Laboratory data, ABPM	17	Stable phase	103	5	Spain	2020	Arévalo-Lorido J C, et al. High Blood Press Cardiovasc Prev 27 (5):399-408.
RCIA	Hierarchical clustering	Patient background, Laboratory data	10	Stable phase	907	4	Spain	2020	Arévalo Lorido J C, et al. Rev Clin Esp (Barc) 220 (7):409-416.
University Hospital, Wroclaw, Poland	Hierarchical clustering	Laboratory data, Echocardiography	7	Stable phase	177	2	Poland	2019	Przewlocka-Kosmala M, et al. J Am Soc Echocardiogr 32 (5):604-615.e606.
Ex-DHF-P	Hierarchical clustering	Patient background, Laboratory data, Echocardiography, Electrocardiogram, Cardiopulmonary exercise testing	324	Stable phase	64	2	Germany	2019	Bahls M, et al. J Clin Med 8 (5).
Northwestern University HFpEF Program	Hierarchical clustering	Patient background, Laboratory data, Echocardiography, Electrocardiogram	46	Stable phase	397	3	US	2015	Shah S J, et al. Circulation 131 (3):269-279.
I-PRESERVE	Latent class analysis	Patient background, Laboratory data	11	Stable phase	4113	6	Multinational	2015	Kao D P, et al. Eur J Heart Fail 17 (9):925-935.

Abbreviations: UK, United Kingdom; US, United states; ABPM, ambulatory blood pressure monitoring

Figure S1. Bayesian information criterion

The plots illustrate the association between the Bayesian information criterion (BIC) and number of groups defined by the latent class analysis. In this analysis, we observed a trend where the BIC decreased with an increasing number of clusters. However, with more groups, the clustering becomes less practical for everyday clinical use and interpreting the characteristics of each group becomes challenging. Using the elbow method, we determined that clustering into 5 groups strike a balance, offering an optimal number of clusters for both practical application and interpretability.

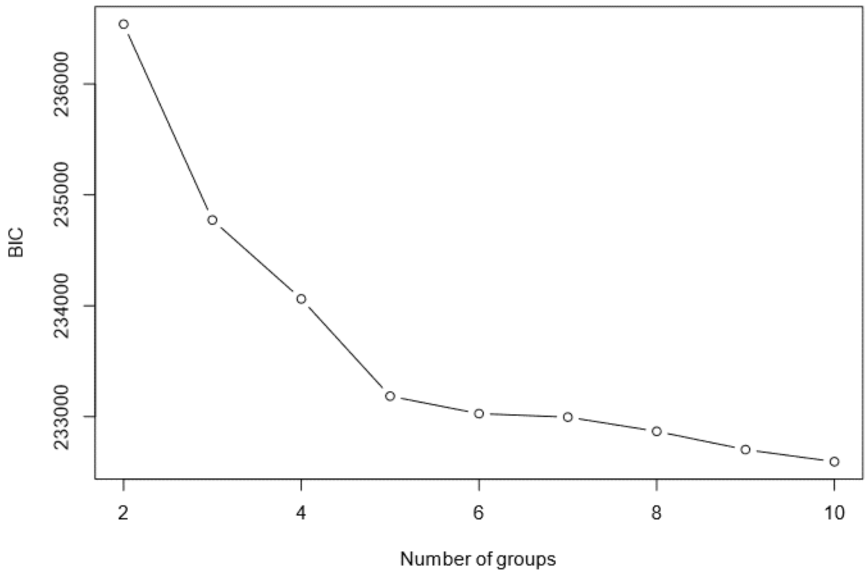

Figure S2. Probabilities of misclassification

Probability of misclassification in the classified groups by latent class analysis in the derivation cohort is summarized in bar graphs. X axis indicates probabilities of misclassification, while Y axis shows the number of cases.

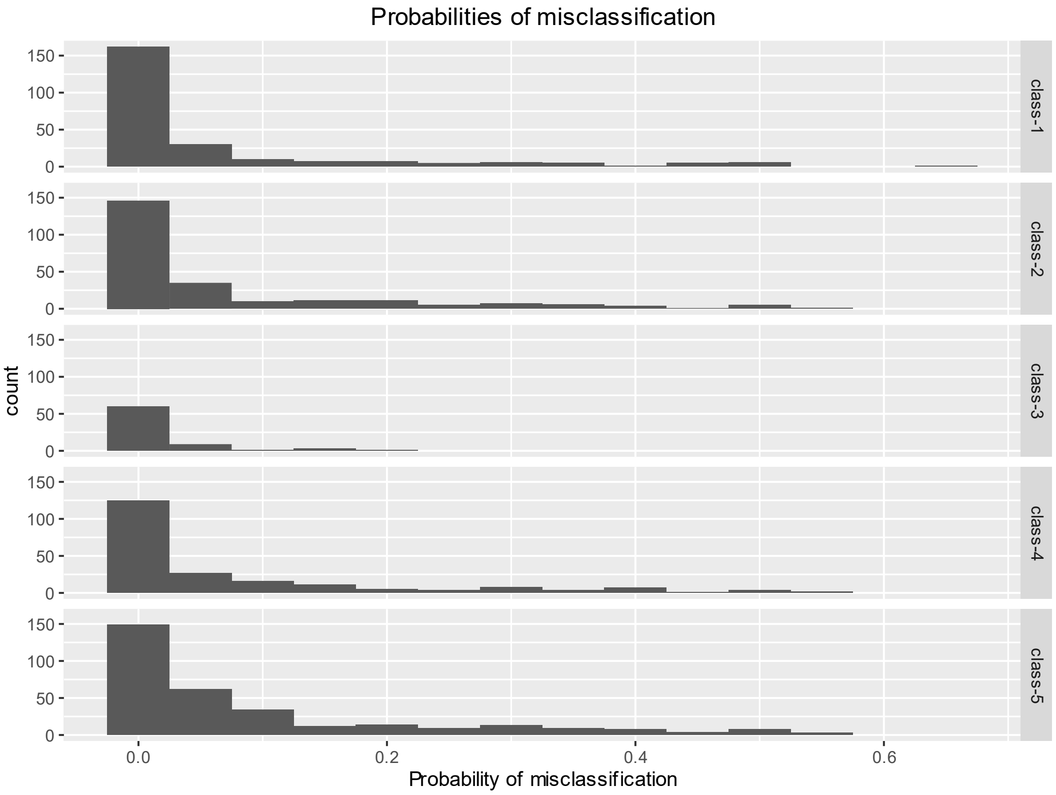

Figure S3. Kaplan-Meier analysis of long-term outcomes based on acute to stable-phase phenotypic progression

We assessed long-term outcomes stratified by the trajectories from acute-phase phenotype to stable-phase phenotype. Panels show the Kaplan Meier survival curves stratified by stable-phase phenotypes in (A) acute-phase phenotype 1, rhythm trouble, (B) acute-phase phenotype 2, ventricular-arterial uncoupling, (C) acute-phase phenotype 3, low output and systemic congestion, and (D) acute-phase phenotype 4, systemic failure.

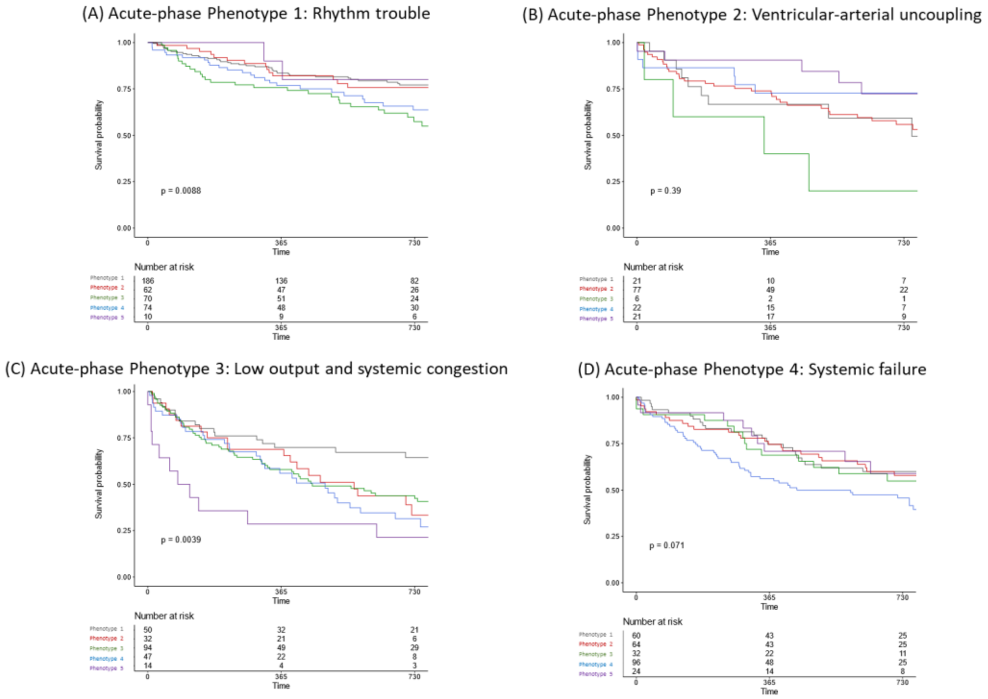

Figure S4. The methods and Prisma diagram of systematic review

<u>**Search code**</u>

((“heart failure with preserved ejection fraction”) OR (“HFpEF”) OR (Heart Failure, Diastolic[MeSH Terms]) OR (Heart Failure, Preserved Ejection Fraction[MeSH Terms]) OR (Heart Failure, Normal Ejection Fraction[MeSH Terms])) AND ((“phenomapping”) OR (“clusters”) OR (“phenogroups”) OR (cluster analysis[MeSH Terms]) OR (cluster analyses[MeSH Terms]) OR (machine learning[MeSH Terms]) OR (“phenotyping”))

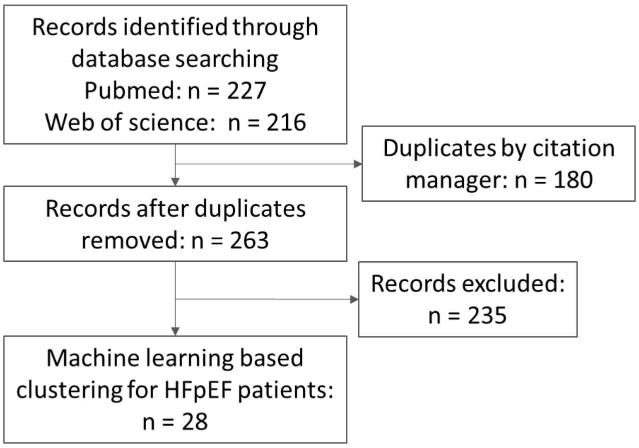

Supplement: Supplementary file 1 — Data S1 Tables S1–S4 Figures S1–S4 [file JAH3-14-e037567-s001.pdf]
